# Data Driven Cell Cycle Model to Quantify the Efficacy of Cancer Therapeutics Targeting Specific Cell-Cycle Phases From Flow Cytometry Results

**DOI:** 10.3389/fbinf.2021.662210

**Published:** 2021-04-27

**Authors:** David W. James, Andrew Filby, M. Rowan Brown, Huw D. Summers, Lewis W. Francis, Paul Rees

**Affiliations:** ^1^ Medical School, Swansea University, Swansea, United Kingdom; ^2^ Flow Cytometry Core Facility and Innovation, Methodology and Application Research Theme, Biosciences Institute, Faculty of Medical Sciences, Newcastle University, Newcastle upon Tyne, United Kingdom; ^3^ College of Engineering, Swansea University, Swansea, United Kingdom

**Keywords:** cell-cycle, cancer, therapeutic, model, differential-evolution, flow-cytometory

## Abstract

Many chemotherapeutic drugs target cell processes in specific cell cycle phases. Determining the specific phases targeted is key to understanding drug mechanism of action and efficacy against specific cancer types. Flow cytometry experiments, combined with cell cycle phase and division round specific staining, can be used to quantify the current cell cycle phase and number of mitotic events of each cell within a population. However, quantification of cell interphase times and the efficacy of cytotoxic drugs targeting specific cell cycle phases cannot be determined directly. We present a data driven computational cell population model for interpreting experimental results, where in-silico populations are initialized to match observable results from experimental populations. A two-stage approach is used to determine the efficacy of cytotoxic drugs in blocking cell-cycle phase transitions. In the first stage, our model is fitted to experimental multi-parameter flow cytometry results from untreated cell populations to identify parameters defining probability density functions for phase transitions. In the second stage, we introduce a blocking routine to the model which blocks a percentage of attempted transitions between cell-cycle phases due to therapeutic treatment. The resulting model closely matches the percentage of cells from experiment in each cell-cycle phase and division round. From untreated cell populations, interphase and intermitotic times can be inferred. We then identify the specific cell-cycle phases that cytotoxic compounds target and quantify the percentages of cell transitions that are blocked compared with the untreated population, which will lead to improved understanding of drug efficacy and mechanism of action.

## Introduction

The use of flow cytometry and more recently, imaging flow cytometry are well established as methods for investigating single cell properties and proliferation in large cell populations (Vermeulen et al., 2003; Darzynkiewicz et al., 2004; Filby et al., 2011). These multi-parameter flow cytometry techniques are particularly useful in assessing the effectiveness of therapeutics which inhibit progress through the cell cycle (Vermeulen et al., 2003). Staining asynchronously dividing cells using CellTraceViolet (CTV), Propidium Iodide (PI) and phospho-histone H3 (pH3) staining (Filby et al., 2011) allows the determination of cell numbers in G_1_, S, G2 and M phases and the number of division rounds each cell has undergone. When a therapeutic agent is introduced *in-vitro*, the change cell population percentages in each phase at a given time point, can indicate which phase transitions are inhibited. However, quantifying the effectiveness of therapeutics is complicated by the complex dynamics and heterogeneity within a cell population. Certainly, the asynchronous nature of a proliferating cell line means that while two cells are in the same cell cycle phase, they may exist in different rounds of division and thus are temporally distinct form one another. This can have a significant impact on interpreting data from cell cycle inhibitory compounds as both cell cycle phase and cell division history, may determine therapeutic response (Filby et al., 2016).

A range of mathematical and computational models have been developed to predict the dynamics of the cell cycle, both for wild type populations and populations treated with therapeutics. Models fall into two categories; explanatory models designed around specific experiments and used to interpret the results or predictive models formulated based on theoretical cell cycle hypothesis (Fuß et al., 2005).

Both types of model rely on an underlying hypothesis and mathematical formulations. Two commonly used mathematical schemes are continuous systems of ODE’s (Sible and Tyson, 2007; Novak et al., 2009) and discrete Boolean models (Davidich and Bornholdt, 2008; Davidich and Bornholdt, 2013; Fauré et al., 2006). While these models are effective in capturing wildtype behavior of specific cell populations and the effect of modulation of modeled regulatory systems, they are often found to be too simplistic to capture the intrinsic processes of the cell cycle (Chaffey et al., 2014; Fuentes-Garí et al., 2015).

Population balance models (PBM) based on systems of first order PDE’s, Faraday et al. (2001) and Basse and Ubezio (2007) are common in the literature. The population of cells in each phase are modeled by continuous population density functions. The number of cell cycle phases modeled varies dependent on the application (Abroudi et al., 2015). The rates of cells entering and exiting each phase are governed by transition functions, which are dependent on cell properties, such as cell DNA content (Liu et al., 2007), mass (Mantzaris et al., 1999), age (Liou et al., 1997; Chaffey et al., 2014), cell size (Liu et al., 2007; Chapman et al., 2008) and cyclin content (García Münzer et al., 2014), either individually or in combination. From a modeling perspective, age-structured models are advantageous as without defining a maximum phase time, cells may remain within a phase indefinitely (Chaffey et al., 2014). However, they can be difficult to verify as cell age is difficult to measure experimentally, compared with more easily observable parameters such as DNA content (Liu et al., 2007).

In contrast to the continuous population distributions utilized in PBMs, cell ensemble models (CEM) model the population as an ensemble of individual cells (Henson, 2005). Simulating cells discretely allows heterogeneity of key cellular parameters across the population to be captured. Altinok et al. (2011) develop a stochastic CEM to study the desynchronization of cell populations and entrainment into the circadian clock. The effects of changing mean interphase times and variability are analyzed. While similar in structure to the model presented herein, Altinok et al. (2011) do not compare directly to experimental data.

Cadart et al. (2018) develop a mathematical frame work to investigate the relative contributions of growth rate and cell cycle duration modulation to mammalian cell size homeostasis. Linear regression is used to fit the growth rate and duration modulation coefficients to experimental data in a balance equation. Designing the model and experiment in tandem is clearly advantageous to confirm model accuracy and identifying key parameters pertaining to cell population growth.

Many models attempt to quantify the efficacy of cancer therapeutics and determine optimal treatment plans. Fuentes-Garí et al. (2015) compare three different cell cycle models to evaluate their suitability for modeling the effect of chemotherapy treatments on leukemia cell populations, demonstrating the difficulty of selecting models that accurately replicates experimental results while producing meaningful biological quantities.

The mode of action of therapeutics often modifies processes only during certain phases of the cell cycle e.g., DNA synthesis (Schwartz and Dickson, 2009). Therefore, to assess the efficacy of the intervention it is essential to determine at what points in the cell cycle the cells are affected. Given the number cells present in all phases in an asynchronous cell population and the heterogeneity of the timings of cell cycle transition processes it is difficult to determine and quantify which phases have been affected.

In previous work we have shown the effectiveness of explanatory stochastic data driven models to assess the distribution of quantum dots markers in asynchronously dividing cell populations, where more traditional statistical analysis has been unsuccessful (Brown et al., 2010; Errington et al., 2010). Virtual in-silico cell populations, containing large numbers of cells, are initialized and optimized to match experimentally determined phase populations. These models capture the variability and heterogeneity of individual cell cycles and events, giving greater insight into population dynamics than population statistics approaches.

In this work we present an explanatory virtual in-silico cell population cell cycle model. The model is used as an analysis tool to extract information from imaging flow cytometry experimental data, extracting inter-phase and inter-mitotic times and quantifying the efficacy of therapeutics in inducing arrest in cell cycle phases.

The model consists of two main steps. The first step requires experimental data from untreated cell populations to determine cell-cycle parameters defining distributions governing the time that cells reside in each cell-cycle phase. The parameters values are obtained by fitting the measured cell precursor frequency (cpF) of the experimental data to the cpF of the virtual population. The second step uses experimental data from the same population of cells plus the addition of a therapeutic after a set number of hours after at the initiation of the experiment. The parameters governing inter-phase time from the untreated populations are used in the model for the treated population. To account for therapeutic induced cell-cycle-phase arrest a blocking probability is assigned to each cell cycle phase, which gives the probability that any attempted transition to the next cell cycle phase is blocked. The values of the blocking parameter are determined by fitting the cpF of the treated in-silico population to the treated experimental population.

We demonstrate the effectiveness of this approach using 4 different cytotoxic agents known to target specific cell cycle phases. The model identifies the cell cycle phases targeted by the agents and quantifies the percentage of transitions blocked in each phase compared to the untreated population.

## Methods

### Experiment

**FIGURE 1:**
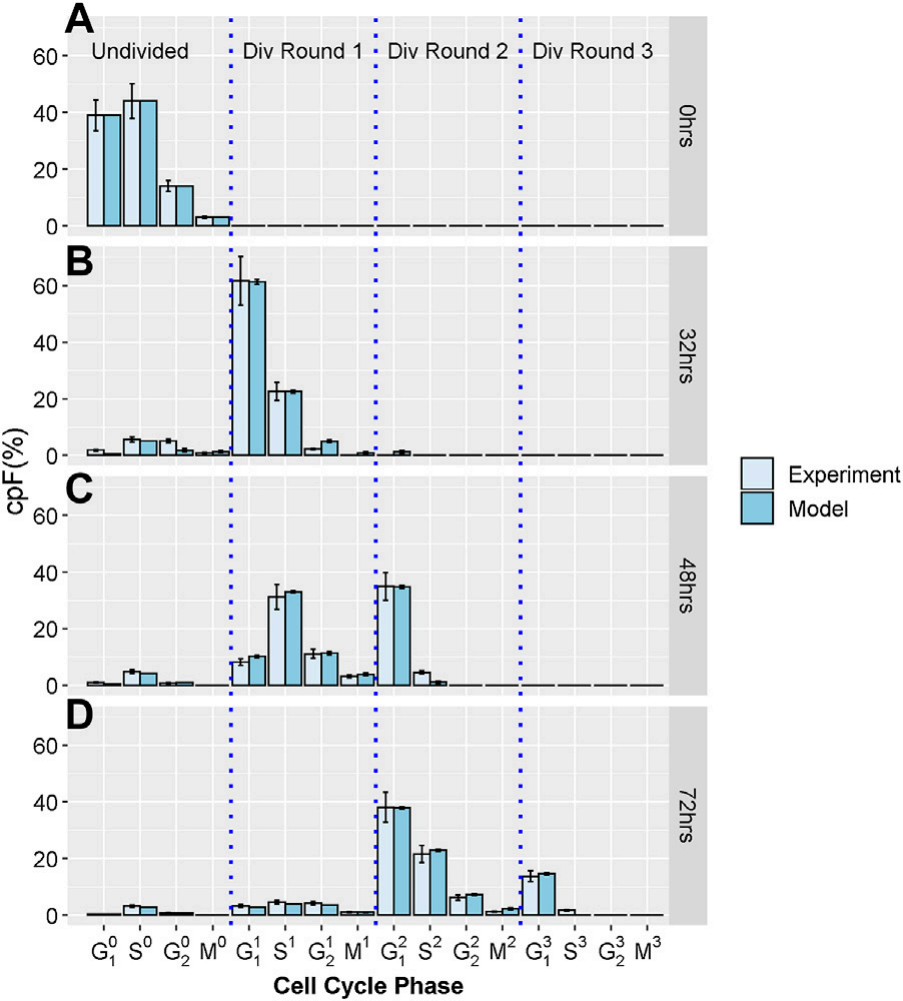
Experimental and model cpF for untreated sample. Light blue bars show cpF from experimental population and dark blue bars show cpF from in-silico population at **(A)** 0 h, **(B)** 32 h, **(C)** 48 h, and **(D)** 72 h (note at 0 h cpF of in-silico population is matched to experimental population). Error bars on experimental data show the largest percentage error for any population observed in the control data. Error bars on model data show standard error from fits of 100 in-silico cell populations.

To generate the experimental dataset, we perform imaging flow-cytometry measurements on populations of T-lymphocyte cells, from the Jurkat cell-line. Cells were stained using CellTraceViolet (CTV), Propidium Iodide (PI) and phospho-histone H3 (pH3) to determine the number of cells in G_1_, S, G_2,_ and M phases. Using a gating strategy previous defined (Begum et al., 2013) we determine the cumulative precursory frequency (cpF), defined as the percentage of the total cell population in each cell cycle phase for each division round. Successive rounds are normalized by a factor of 2^n^ where n is the division round, *n* = 0 corresponding the initial undivided population. For further experimental details see supplementary information. Figure 1 shows experimental cpF’s for an untreated Jurkat cell population measured at times T = 0, 32, 48 and 72 h, generated by the methods discussed above and in our previous work (Barteneva et al., 2016). It is evident that cells become increasingly dispersed throughout different phases and division rounds as measurement T increases.

Using the same methodology, further experiments were performed where a cytotoxic drug is introduced to cell populations after 32 h. The cpF is measured at the time the drug is added and again at 48 h. Four different therapeutic treatments were used; Nocodazole, Demecolcine, 5-Fluorouracil, and Etoposide.

All experiments were carried out in triplicate and the mean and standard deviation of the proportion of cells in each phase calculated. As with previous work (Begum et al., 2013; Filby et al., 2011) we observed very small errors, especially with the shorter cell cycle phases, therefore we choose to take a pessimistic approach and assume an error for each phase equal to the largest percentage error observed which was 14% (Figure 1). However, we note that the least squares cost function for fitting the model is robust to the inclusion of Gaussian errors (especially small and uncorrelated errors observed in these experiments) which makes this approach easily applicable.

### Model

To determine cell phases targeted by each therapeutic, we have developed a transient cell cycle model utilizing a discrete in-silico cell population. The model was developed using MATLAB (Mathworks Inc., 2016). Each cell in an in-silico is represented by three parameters; i) its current cell-cycle phase (G_1_, G_2_, S or M), ii) the time it has resided in the current cell cycle phase, *t,* and iii) the number of division rounds the cell has undergone, *N* (undivided cells are labeled as division round 0). The transition of cells to their next cell-cycle phase is a stochastic process, governed by time dependent probability distribution functions (PDF), $f_{p}^{N}\left(t\right)$ (where subscript *p* denotes the cell-cycle phase). In this work, generalized extreme value (GEV) distributions are used to define the cell cycle PDFs. GEV distributions are characterized by the location parameter $\mu_{p}^{N}\;$ , scale parameter $\sigma_{p}^{N}$ and the shape parameter ξ . The shape parameter was fixed at 0.68 for all PDF’s, resulting in a long-tailed distribution. The chosen distribution is appropriate as cell-cycle phase transitions are intrinsically long-tailed (Brown et al., 2010; Errington et al., 2010). Furthermore, the use of extreme value distributions as a suitable parameterizable model for S-phase inter-phase times have been demonstrated previously (Zhang et al., 2017). To assess the effect of drug treatment an additional routine is incorporated into the model, where each cell cycle phase is assigned a division round independent blocking factor $b_{p}$ between 0 and 1. The blocking parameter represents the probability that an attempted phase transition is blocked due to the therapeutic.

#### Initialization

For each run of the model, an in-silico population of 20,000 individual cells is initialized with cpF matched to experimental data at time T =0 . The values of *t* for each cell in the experimental population cannot be obtained from the experimental data. Therefore, for each cell, *t* is drawn randomly from a uniform distribution, lower bounded by 0 and upper bounded by a predefined limit which is equal to the upper limit set on $\mu_{p}^{N}$ for the fitting routine (see Supplementary Table S3).

#### Transition Through Cell Cycle

Starting at T =0 , the model is incremented through time with time step δT . At each time step, *t* is incremented by δT for each cell. The probability of it having transitioned between phases since the last time step, is evaluated using the Hazard function, defined as

$$P_{p}^{N}\left(\delta T,t\right)=\frac{F_{p}^{N}\left(t+\delta T\right)-F_{p}^{N}\left(t\right)}{1-F_{p}^{N}\left(t\right)}$$ (1)

where $\;F_{p}^{N}\left(t\right)$ is the cumulative distribution function (CDF) of $f_{p}^{N}\left(t\right)$ . GEV CDF’s were generated using the MATLAB gevcdf function (Mathworks Inc., 2016). The Hazard function gives the probability of a cell transitioning into the next phase of its cell cycle between time points *t* and t+δt , given that it has not transitioned previously. To assess phase transitions in the current time-step, a uniform random number between 0 and 1 is generated for each cell. If the random number of a cell is less than $P_{p}^{N}\left(\delta t,t\right)$ , a phase transition occurs, the cell’s phase is updated and *t* is set to 0. If a cell transitions from the M phase to the G_1_ phase the number of division rounds it has undergone is incremented by 1 and a daughter cell, with parameters set equal to the original cell, is added to the population, representing mitotic events.

By using GEV PDF’s with ξ>0 , the maximum probability of transition occurs when $t=\mu_{p}^{N}$ (see Supplementary Figure S3). For $t>\mu_{p}^{N}$ , the probability of transition to the next phase decreases with T , so the longer a cell resides within a cell-cycle phase the less likely it becomes to exit the phase in the next time step. When a cell enters a phase, there is a finite time for which $P_{p}^{N}=0$ , so cells must reside in a cell phase for some finite duration before exiting, as would be the case for physical cell populations (see Supplementary Material for further details). If the blocking routine is used, a second uniform random number between 0 and 1 is generated for any cell due to undergo phase transition. If this random number is lower than $b_{p}$ for the cells current phase, the transition is blocked. The cell remains in its current phase and *t* is incremented by δT . A complete ordered list describing the iterative steps of the model is given in supplementary information.

#### Fitting to Experimental Data

##### Model Applied to Untreated Population

To obtain optimal values of $\mu_{p}^{N}$ and $\sigma_{p}^{N}$ , for each cell-cycle phase, we fitted the cpF of in-silico cell populations to the untreated experimental populations. A differential evolution (DE) algorithm (Storn and Price, 1997), was used to minimize the sum of squares between experimental and model cpF’s, at the experimental measurement timepoints of 32, 48, and 72 h, with $\mu_{p}^{N}$ and $\sigma_{p}^{N}$ as fit parameters. Upper and lower limits on the range of values which the fitting parameters were permitted to take are defined in Supplementary Table S3. During fitting of the untreated population, models were run over 200 time-steps for a virtual period of 72 h (δT=0.36 h) . A full description of the fitting scheme is given in Supplementary Material.

Initially, we used separate values of $\mu_{p}^{N}$ and $\sigma_{p}^{N}$ for each division round, resulting in 2×4×N fitting parameters. We take N as 4, as there are no cells in division rounds higher than 3 for any of the experimental datasets, resulting in 32 fit parameters. It was found that the standard deviation of the G_2_ and M phases did not affect the goodness of fit measurements significantly, so these were fixed at 0.1, resulting in 24 fit parameters. The resulting fits are shown in Supplementary Figure S2. Fitting the data in this way showed similar fit values for G_1_ and S in division rounds 1 and 2, whereas G_2_ and M had similar fitted values across all division rounds (see Supplementary Figure S3).

Given the similarities of the fit parameters across different division rounds, the number of fit parameters was reduced by using the same values of $\mu_{p}^{N}$ and $\sigma_{p}^{N}$ for multiple division rounds. For phases G_2_ and M, $\mu_{G_{2}}^{0}=\mu_{G_{2}}^{1}=\;\mu_{G_{2}}^{2}=\mu_{G_{2}}^{3}$ and $\mu_{M}^{0}=\mu_{M}^{1}=\;\mu_{M}^{2}=\mu_{M}^{3}$ . For G_1_ and S phases, $\mu_{G_{1}}^{1}=\mu_{G_{1}}^{2}=\;\mu_{G_{1}}^{3}\neq \;\mu_{G_{1}}^{0}$ , $\sigma_{G_{2}}^{1}=\sigma_{G_{2}}^{2}=\sigma_{G_{2}}^{3}\neq \sigma_{G_{2}}^{0}$ , $\mu_{S}^{1}=\mu_{S}^{2}=\mu_{S}^{3}\neq \;\mu_{S}^{0}$ and $\sigma_{S}^{1}=\sigma_{S}^{2}=\sigma_{S}^{3}\neq \;\sigma_{S}^{0}$ . This reduces the final number of fit parameters to 10.

In the experimental cpF plots it is evident that a small percentage of cells are arrested in division round 0 for the duration of the experiment (see Figure 1, panels C and D). These arrested cells are considered within the model, by randomly selecting a subset of cells from the initial population that cannot transition from their original phase or division round. The cpF of the total population of in-silico cells arrested is matched to the experimental data.

##### Model Applied to Treated Population

To quantify the effect of therapeutics on cell cycle progression, we fitted the cpF of in-silico cell populations to the treated experimental populations, using the DE algorithm (Storn and Price, 1997). The values of $\mu_{p}^{N}$ and $\sigma_{p}^{N}$ were fixed to the values obtained from the untreated fits, and the phase transition blocking probabilities, $b_{p}$ , were the fit parameters.

In the experiments, therapeutics were added at 32 h. Correspondingly, for *T* < 32 h, we set $b_{p}=0$ for all cell cycle phases, before assigning a value between 0 and 1 for *T* > 32°h. At 72°h significant amounts of cell death was observed in the experimental population. As the current model does not account for cell death, fits of the model cpF to experiment were only performed at 48°h. We performed fits for four cytotoxic drugs, Nocodazole, Demecolcine, 5-Fluorouracil, and Etoposide.

## Results

### Untreated Population

Figures 1A-D show cpF's of model and experimental populations at 0, 32, 48 and 72 hours respectively. There is good correspondence between the cpF’s of the model and experimental data for all cell cycle phases and division rounds. The final values of $\mu_{p}^{N}$ and $\sigma_{p}^{N}$ were obtained by averaging over 100 individual fits of the data (see Supplementary Material). The fitted values of $\mu_{p}^{N}$ and $\sigma_{p}^{N}$ for each cell cycle phase and IMT are shown Table 1. Since $\mu_{p}^{N}$ represents the time of maximum transition probability of a cell exiting a cell-cycle phase, we interpret $\mu_{p}^{N}$ as the interphase times and IMT as the sum of $\mu_{p}^{N}$ over each division round.

**TABLE 1:** Mean vaues of transition PDF location, *µ*, and scale parameters, *σ*, for final untreated model, averaged over 100 individual fits.

Division round	μ G_1_ (h)	μ S (h)	μ G_2_ (h)	μ M (h)	*σ* G_1_ (h)	*σ* S (h)	Intermitotic time (h)
0	7.11 (±0.65)	14.76 (±0.17)	4.84 (±0.18)	1.69 (±0.21)	0.18 (±0.13)	0.37 (±0.19)	28.41 (±0.73)
1, 2	14.76 (±0.13)	8.14 (±0.2)			2.33 (±0.20)	3.91 (±0.79)	29.43 (±0.37)

The time of transition through division round 0 is expected to be shorter than for other division rounds, as cells in the experimental are not synchronized and already part way through their current cell cycle. For G_1_ phase, $\mu_{G_{1}}^{0}$ is 7.1 h, compared with 14.8 h for $\mu_{G_{1}}^{1,2,3}$ , corresponding to the expected behavior. However, for S phase $\mu_{S_{1}}^{0}$ is 14.8 h for division round 0, significantly longer than the 8.1 h for $\mu_{S_{1}}^{1,\;2,3}$ . The reason for the discrepancy in parameters between division round 0 and rounds 1 and 2 is due to the random initialization of cell interphase times (this is required by the model because in this first measurement we have no reference to the amount of time that a cell has spent in its current phase). Division round 0 allows the model to adjust to account for the discrepancy between the randomly initialized model phase times and the unknown experimental phase times. Parameters obtained for proceeding division rounds represent the steady state solution, confirmed by the similarity in results obtained from division rounds 1 and 2 (see Supplementary Figure S3). Therefore, parameters from division round 0 should not be taken to represent the experimental population. We obtain an intermitotic time (IMT) of 29.43 h for division rounds 1 and 2. From the experimental cpF data is it clear that the mean IMT of the experimental population is >24 h, as most cells are in division round 2 at 72 h.

### Treated Population

**FIGURE 2:**
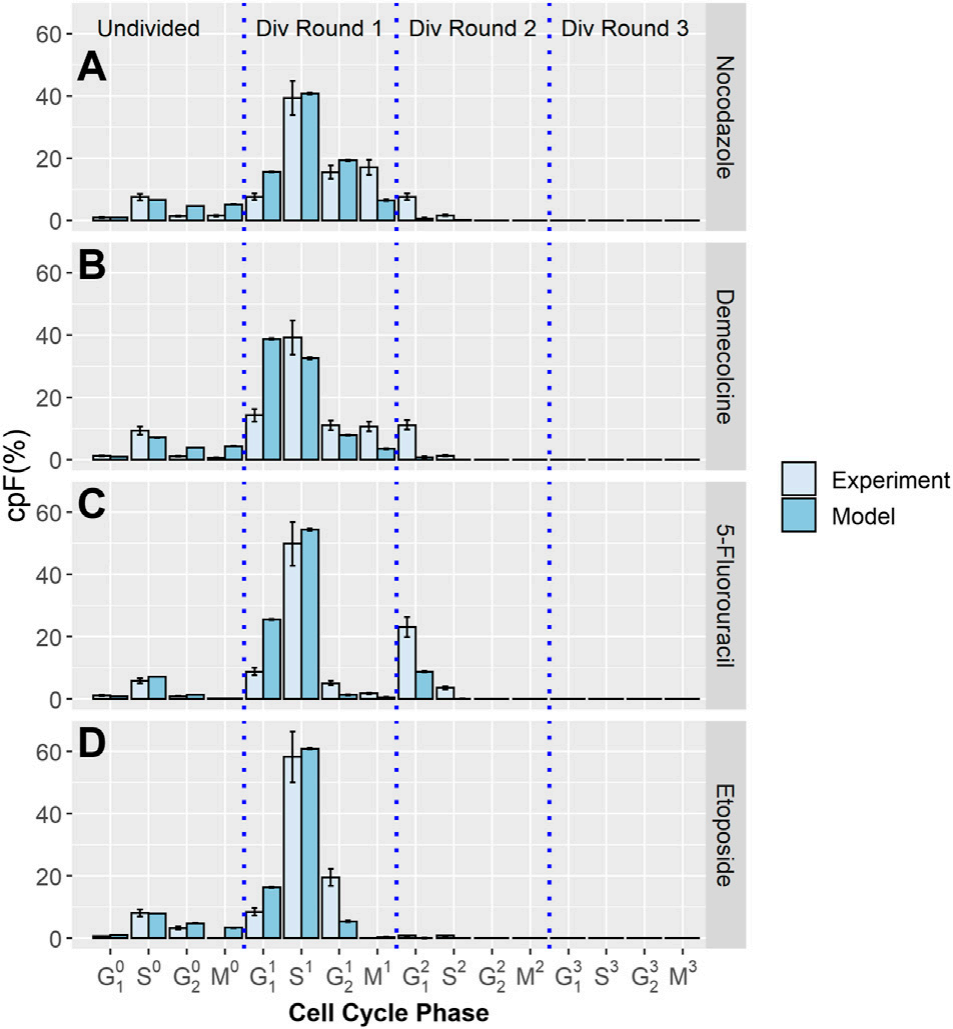
Experimental and model cpF results for cell populations treated with different inhibitor drugs. Experimental (light blue bars) and model (dark blue bars) cpFs after 48 h, after introducing therapeutics; **(A)** Nocodazole **(B)** Demecolcine **(C)** 5-Fluorouracil **(D)** Etoposide at 32 h. Error bars on experimental data show the largest percentage error for any population observed in the control data. Error bars on model data show standard error from fits of 100 in-silico cell populations.

The cpF of in-silico populations fitted to experimental data after 48 h for the four therapeutics, Nocodazole, Demecolcine, 5-Fluorouracil, and Etoposide is shown in Figure 2. There is clear correspondence between model and experimental cpF’s. The assumption that the phase transition PDF’s of the untreated population can be used for the treated populations is shown to be reasonable given the quality of the fits of the in-silico populations cpF to experiment (Figure 2). The blocking factors for each phase gives a measure of the probability of a cell attempting to transition into its next phase being blocked. If a transition is unsuccessful the phase time *t* is not reset and it is still able to attempt a transition in a later time step. This approach is designed to capture delay in transitions due to the therapeutic, rather than explicitly capturing cell cycle arrest. However, due to the use of GEV PDF’s (with ξ>0 ), the blocking parameter can limit transitions when $P_{p}^{N}$ peaks, pushing *t* to a regime where $P_{p}^{N}$ tends to 0, implicitly applying cell arrest.

**FIGURE 3:**
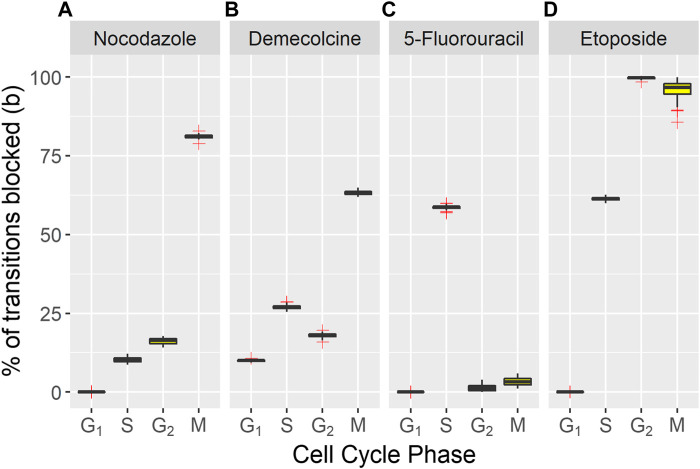
Percentage of transitions blocked from each cell phase of treated cell populations compared with the untreated population. Box plots show median (red horizontal line), interquartile range (blue boxes), range of data points within a factor of 1 of the interquartile range (whiskers) and outliers (red crosses) of the blocking factors for **(A)** Nocodazole **(B)** Demecolcine **(C)** 5-Fluorouracil **(D)** Etoposide blocking drugs.

The values of $b_{p}$ for each drug in each phase, averaged over 100 fits are shown in Figure 3 (also see Supplementary Table S1). For each treatment the cell-cycle phases targeted correspond to known behaviour. Figure 3A) shows the percentage of transitions blocked by nocodazole treatment. Nocodazole is a antineoplastic agent which interferes with the polymerization of microtubules. Treated cells are arrested in the G_2_ and M phases (Ben-Ze’ ev et al., 1979; Blajeski et al., 2002). This behavior is reflected in the model population, with 81.1% of transitions from M phase and 16.2% of transitions from G_2_ blocked.

Figure 3B shows the percentage of transitions blocked for Demecolcine treatment. Demecolcine depolymerizes microtubules and limits microtubule formation arresting cells in the M phase. This depolymerization effect can also cause the breakdown cytoskeletal structures resulting in arrest in other phases (Mukhtar et al., 2014). This behavior is reflected in the blocking percentages of each phase with 63.2% blocked on the M phase and smaller but high percentages blocked in the other three phases (see Supplementary Table S5).

Figure 3C the shows percentage of transitions blocked for 5-Fluorouracil treatment. 5-Fluorouracil is principally a thymidylate synthase inhibitor, blocking synthesis of pyrimidine thymidine and in consequence disrupting DNA replication (Miura et al., 2010). Cells are predominately arrested in the S phase. This is reflected by the model with 58.7% of transitions from the S phase blocked, while the percentage of transitions blocked in the other phases are negligible.

Figure 3D shows the percentage of transitions blocked for Etoposide treatment. Etoposide inhibits DNA re-litigation (Montecucco et al., 2015), causing apoptosis of cells in the S phase. Errors in the DNA due to a sub-optimal re-litigation process can result in arrest in other phases, particularly at the G_2_ phase DNA damage checkpoint (Nam et al., 2010). The model shows that 61.3% of transitions from the S phase are blocked, 99.7% of transitions from the G_2_ phase are blocked. This indicates that even if re-litigation does occur the damage caused to DNA by Etoposide is too significant to pass the DNA checkpoint for all effected cells.

The phases targeted by each inhibitor drug predicted by the model correspond well with expected behavior, indicating that the model is successfully capturing the dynamics of the cell population and the effect of blocking drugs. The efficacy of inhibitor drugs is quantified using a simple and easily interpretable parameter $b_{p}$ .

## Discussion

Extraction of population wide statistical quantities from cell proliferation experiments is difficult due to heterogeneity and limitations of measurement techniques. Explanatory models can be used to extract information from experimental data sets which cannot be obtained directly from measurements.

In this work we present an explanatory modeling approach which is used as a data analysis tool to quantify the efficacy of therapeutics which cause arrest in specific cell-cycle phases. We validate the modeling approach using four different therapeutics on cpF data from imaging flow cytometry experiments on the Jurkat cell line. For both treated and untreated populations, the predicted cpF’s of the in-silico population closely match experimental population at the measurement time points. The model accurately identifies the cell cycle phases targeted by each therapeutic. In addition, the efficacy of the treatment in arresting cells in each cell-cycle phase is quantified by the percentage of phase transitions blocked compared with the untreated population.

For compounds with unknown mechanisms of action or drug combination therapies, this information provides far greater insight into how cell-cycle phases are targeted when compared with simply assessing the difference in accumulation of cells in different cell-cycle phases in treated and untreated samples. Assessing targets based on accumulation alone may ignore subtle effects where a high percentage of cells being arrested in one phase masks arrest events in other phases. This could be particularly important for setting optimal does in combination therapies where multiple phases are targeted by the different treatments.

## Data Availability

The original contributions presented in the study are included in the article/Supplementary Material, further inquiries can be directed to the corresponding authors.

## References

[B1] Abroudi A. Samarasinghe S. Kulasiri D. (2015). A Review of Computational Models of Mammalian Cell Cycle. 564-570. Retrieved from http://www.mssanz.org.au/modsim2015/C2/abroudi2.pdf .

[B2] Altinok A. Gonze D. Lévi F. Goldbeter A. (2011). An Automaton Model for the Cell Cycle. Interf. Focus. 1 (1), 36–47. 10.1098/rsfs.2010.0009 PMC3262245 22419973

[B3] Barteneva Nantha S. Vorobjev Ivan A. (Editors) (2016). Imaging Flow Cytometry. New York: Springer.

[B4] Basse B. Ubezio P. (2007). A Generalised Age- and Phase-Structured Model of Human Tumour Cell Populations Both Unperturbed and Exposed to a Range of Cancer Therapies. Bull. Math. Biol. 69 (5), 1673–1690. 10.1007/s11538-006-9185-6 17361361

[B5] Begum J. Day W. Henderson C. Purewal S. Cerveira J. Summers H. (2013). A Method for Evaluating the Use of Fluorescent Dyes to Track Proliferation in Cell Lines by Dye Dilution. Cytometry 83 (12), 1085–1095. 10.1002/cyto.a.22403 24166880

[B6] Ben-Ze’ev A. Farmer S. R. Penman S. (1979). Mechanisms of Regulating Tubulin Synthesis in Cultured Mammalian Cells. Cell 17 (2), 319–325. 10.1016/0092-8674(79)90157-0 455467

[B7] Blajeski A. L. Phan V. A. Kottke T. J. Kaufmann S. H. (2002). G1 and G2 Cell-Cycle Arrest Following Microtubule Depolymerization in Human Breast Cancer Cells. J. Clin. Invest. 110 (1), 91–99. 10.1172/jci13275 12093892 PMC151025

[B8] Brown M. R. Summers H. D. Rees P. Chappell S. C. Silvestre O. F. Khan I. A. (2010). Long-term Time Series Analysis of Quantum Dot Encoded Cells by Deconvolution of the Autofluorescence Signal. Cytometry 77A (10), 925–932. 10.1002/cyto.a.20936 21290466

[B9] Cadart C. Monnier S. Grilli J. Sáez P. J. Srivastava N. Attia R. (2018). Size Control in Mammalian Cells Involves Modulation of Both Growth Rate and Cell Cycle Duration. Nat. Commun. 9 (1), 3275. 10.1038/s41467-018-05393-0 30115907 PMC6095894

[B10] Chaffey G. S. Lloyd D. J. B. Skeldon A. C. Kirkby N. F. (2014). The Effect of the G1- S Transition Checkpoint on an Age Structured Cell Cycle Model. PLoS ONE 9 (1), 1–17. 10.1371/journal.pone.0083477 PMC3886982 24416166

[B11] Chapman S. J. Plank M. J. James A. Basse B. (2008). A Nonlinear Model of Age and Size-Structured Populations with Applications to Cell Cycles. ANZIAM J. 49 (2), 151–169. 10.1017/S144618110001275X

[B12] Darzynkiewicz Z. Crissman H. Jacobberger J. W. (2004). Cytometry of the Cell Cycle: Cycling through History. Cytometry 58A (1), 21–32. 10.1002/cyto.a.20003 14994216

[B13] Davidich M. I. Bornholdt S. (2008). Boolean Network Model Predicts Cell Cycle Sequence of Fission Yeast. PLoS One 3 (2), e1672. 10.1371/journal.pone.0001672 18301750 PMC2243020

[B14] Davidich Maria. I. Bornholdt S. (2013). Boolean Network Model Predicts Knockout Mutant Phenotypes of Fission Yeast. PLoS ONE 8 (9), e71786. 10.1371/journal.pone.0071786 24069138 PMC3777975

[B15] Dickson M. A. Schwartz G. K. (2009). Development of Cell-Cycle Inhibitors for Cancer Therapy. Curr. Oncol. 16 (2), 36–43. 10.3747/co.v16i2.428 PMC2669234 19370178

[B16] Errington R. J. Brown M. R. Silvestre O. Njoh K. L. Chappell S. C. Khan I. (2010). Single Cell Nanoparticle Tracking to Model Cell Cycle Dynamics and Compartmental Inheritance. Cell Cycle 9 (1), 121–130. 10.4161/cc.9.1.10246 20016285

[B17] Fauré A. Naldi A. Chaouiya C. Thieffry D. (2006). Dynamical Analysis of a Generic Boolean Model for the Control of the Mammalian Cell Cycle. Bioinformatics 22 (14), e124–31. 10.1093/bioinformatics/btl210 16873462

[B18] Filby A. Day W. Purewal S. Martinez-Martin N. (2016). “The Analysis of Cell Cycle, Proliferation, and Asymmetric Cell Division by Imaging Flow Cytometry,” in Imaging Flow Cytometry: Methods and Protocols. Editors Barteneva, N. S. Vorobjev I. A. (New York, NY: Springer New York), 1389, 71–95. 10.1007/978-1-4939-3302-0_5 27460238

[B19] Filby A. Perucha E. Summers H. Rees P. Chana P. Heck S. (2011). An Imaging Flow Cytometric Method for Measuring Cell Division History and Molecular Symmetry during Mitosis. Cytometry 79A (7), 496–506. 10.1002/cyto.a.21091 21638766

[B20] Fuentes-Garí M. Misener R. Georgiadis M. C. Kostoglou M. Panoskaltsis N. Mantalaris A. (2015). Selecting a Differential Equation Cell Cycle Model for Simulating Leukemia Treatment. Ind. Eng. Chem. Res. 54 (36), 8847–8859. 10.1021/acs.iecr.5b01150

[B21] Fuss H. Dubitzky W. Downes C. S. Kurth M. J. (2005). Mathematical Models of Cell Cycle Regulation. Brief Bioinformatics 6 (2), 163–177. 10.1093/bib/6.2.163 15975225

[B22] García Münzer D. G. Kostoglou M. Georgiadis M. C. Pistikopoulos E. N. Mantalaris A. (2014). A Cyclin Distributed Cell Cycle Model in GS-NS0. Comput. Aided Chem. Eng. 33 (280813), 19–24. 10.1016/b978-0-444-63456-6.50004-1 PMC4344234 25723523

[B23] Henson M. A. (2005). Cell Ensemble Modeling of Metabolic Oscillations in Continuous Yeast Cultures. Comput. Chem. Eng. 29 (3), 645–661. 10.1016/j.compchemeng.2004.08.018

[B24] Liou J.-J. Srienc F. Fredrickson A. G. (1997). Solutions of Population Balance Models Based on a Successive Generations Approach. Chem. Eng. Sci. 52 (9), 1529–1540. 10.1016/s0009-2509(96)00510-6

[B25] Liu Y.-H. Bi J.-X. Zeng A.-P. Yuan J.-Q. (2007). A Population Balance Model Describing the Cell Cycle Dynamics of Myeloma Cell Cultivation. Biotechnol. Prog. 23 (5), 1198–1209. 10.1021/bp070152z 17691814

[B26] Mantzaris N. V. Liou J.-J. Daoutidis P. Srienc F. (1999). Numerical Solution of a Mass Structured Cell Population Balance Model in an Environment of Changing Substrate Concentration. J. Biotechnol. 71 (1–3), 157–174. 10.1016/s0168-1656(99)00020-6

[B27] Matlab 2016b (2016). Natick. Massachusetts, United States: The MathWorks, Inc.

[B28] Miura K. Kinouchi M. Ishida K. Fujibuchi W. Naitoh T. Ogawa H. (2010). 5-FU Metabolism in Cancer and Orally-Administrable 5-FU Drugs. Cancers (Basel) 2 (3), 1717–1730. 10.3390/cancers2031717 24281184 PMC3837334

[B29] Montecucco A. Zanetta F. Biamonti G. Molecolare G. (2015). Review article : Molecular Mechanisms Of Etoposide, EXCLI J. 14: 95–108. 10.17179/excli2015-561 26600742 PMC4652635

[B30] Mukhtar E. Adhami V. M. Mukhtar H. (2014). Targeting Microtubules by Natural Agents for Cancer Therapy. Mol. Cancer Ther. 13 (2). 275–284. 10.1158/1535-7163.MCT-13-0791 24435445 PMC3946048

[B31] Nam C. Doi K. Nakayama H. (2010). Etoposide Induces G2/M Arrest and Apoptosis in Neural Progenitor Cells via DNA Damage and an ATM/P53-Related Pathway. Histol. Histopathol. 25 (4), 485–493. 10.14670/HH-25.485 20183801

[B32] Novak I. L. Kraikivski P. Slepchenko B. M. (2009). Diffusion in Cytoplasm: Effects of Excluded Volume Due to Internal Membranes and Cytoskeletal Structures. Biophysical J. 97 (3), 758–767. 10.1016/j.bpj.2009.05.036 PMC2718152 19651034

[B33] Rees D. B. F. Hayter P. Kirkby N. F. (2001). A Mathematical Model of the Cell Cycle of a Hybridoma Cell Line. Biochem. Eng. J. 7 (1), 49–68. 10.1016/s1369-703x(00)00101-7 11150796

[B34] Sible J. C. Tyson J. J. (2007). Mathematical Modeling as a Tool for Investigating Cell Cycle Control Networks. Methods 41 (2), 238–247. 10.1016/j.ymeth.2006.08.003 17189866 PMC1993813

[B35] Storn R. Price K. (1997). Differential Evolution – A Simple and Efficient Heuristic for Global Optimization over Continuous Spaces. J. Glob. Optimization 11 (4), 341–359. 10.1023/a:1008202821328

[B36] Vermeulen K. Bockstaele D. R. Van. Berneman Z. N. (2003). The Cell Cycle - a Review of Regulation, Deregulation, and Therapeutic Targets in Cancer. Cell Prolif. 36 (3), 131–149. 10.1046/j.1365-2184.2003.00266.x 12814430 PMC6496723

[B37] Zhang Q. Bassetti F. Gherardi M. Lagomarsino M. C. (2017). Cell-to-cell Variability and Robustness in S-phase Duration from Genome Replication Kinetics. Nucleic Acids Res. 45 (14), 8190–8198. 10.1093/nar/gkx556 28854733 PMC5737480

