# Supplementary material for: Data Driven Cell Cycle Model to Quantify the Efficacy of Cancer Therapeutics Targeting Specific Cell-Cycle Phases From Flow Cytometry Results
Source: Front Bioinform. 2021 Apr 27;1:662210. doi: 10.3389/fbinf.2021.662210 (PMC9581040; doi:10.3389/fbinf.2021.662210)
Supplement: Supplementary file 1 [file DataSheet1.docx]

Supplementary Material

## Experimental Method

**Fluorescent dye labelling of viable cells.**

In all experiments, Jurkat cells were labelled with CTV reagent as described previously. Briefly, Jurkat cells (E6.1), maintained in RPMI culture media (Invitrogen, Paisley, UK containing 10% FBS, Penicillin/Streptomycin, Glutamine and 2-Mercaptoethanol) at 37°C/5% CO_2_ were harvested, counted using a Vi-Cell (Beckman Coulter), washed once in serum-free media and re-suspended in Cell Trace Violet labelling solution (CTV, 2 μM in pre-warmed PBS, Invitrogen, A10198) at a density of 4 x 10^6^/ml at 37°C for 30 min. FBS was added (5% v/v) to absorb cell free CTV. After 10 minutes, cells were washed into serum free media for cell sorting. In certain experiments, Jurkats were also labelled with Dye Cycle Ruby (DCR, Invitrogen, A) at 37 deg in order to identify cells with G1, S and G2/M DNA content.

**Cell Sorting**

Cell sorting was performed using a BD FACS Aria I system using a sorting strategy described previously (Filby et al., 2011). Briefly, live cells were identified using PI exclusion in the blue 585/15 channel (x volts), CTV in the Violet 440/40 channel and. Where appropriate, DCR was read using the 695/40 blue filter (volts). Sort channel widths were set using the CV of the CTV labelled population and also the gate channel width reported through the 8-bit channel scaling option in DIVA software. Sorting was performed using the 16-16-0 sort mask and the sort was performed at a flow rate of 6 units with an event rate of approximately 10,000 events per second. Sorted droplets were collected into 15 ml falcon tubes containing 200 ul of FCS. After sorting, a portion of sorted sample was re-run through the machine at the exact same setting and flow rate to measure the channel and CV increase of the post-sort populations. To assess machine-related measurement errors, Violet excited beads (AlignFlow Plus 6 μM, Invitrogen, A7305) were sorted using a single channel width and re-run as described (Filby et al., 2007).

**Cell culture and drug treatment**

Sorted Jurkat cells were resuspended in fresh complete growth medium (RPMI 1640 +10% FBS, 2-Me and Pen Strep) at a density of 1 x 10^6^ /ml. For non-imaging flow cytometry experiments, 100 ul of cells were cultured in 96 well round bottom plate. For IFC experiments, CTV labelled cells were set up in T25 culture flasks. Cells were cultured for different time periods in the presence of either nocodazole (Noc, 0.1 μM, SIGMA Cat), demecolcine (Dcol, 0.01 μM, Sigma Cat), 5-fluorouracil (5-F, 10 μM, Sigma cat), etoposide (Etop, 1 μM, Sigma Cat:) or vinorelbine ditartrate (VB, 1 μM. Sigma Cat:). All doses used were determined by titration with viability and stage-specific cell cycle inhibition as read-outs (Figure S1). In some experiment’s inhibitors were present for the last 16 hours of a 48hr culture. In other experiments, inhibitors were also added to cultures at 32 hrs. and either washed away at 48 hrs. or left on for analysis at 72 hrs. In all cases, untreated and DMSO vehicle treated cells were cultured as a control. At the defined harvest times and prior to drug addition, some cells were checked for viability by PI exclusion and also CTV peak resolution prior to fixation in 70% ethanol for intracellular staining and DNA analysis.

**Cell Staining**

Cells (1 x 10^6^) were seeded in a 96 well round bottom plate, washed once with PBS containing 2% FBS (wash buffer), fixed with 2% formaldehyde (Polysciences, Inc, USA #11814) for 45 minutes at room temperature (RT) then permeabilised with 0.1% Triton-x 100 for 5 min. Samples were stained with mouse anti-pH3 Ser 10 (Cell signalling Inc, #9706S) for 1hr at RT followed 2 washes. Cells were incubated with anti-mouse AF488 (Invitrogen) for 45 min at RT in the dark. Cells were washed prior to acquisition on either an LSRII (BD) or an ImageStream-x system (ISx, Amnis corp., Seattle USA). Single stained controls were prepared for spectral compensation.

**LSRII acquisition and analysis**

Samples were incubated with RNAse A and PI (50 μg/ml) for 30 min at RT. CTV (Ex 405nm Em 440/40), AF488 (Ex 488nm Em 530/30) PI (Ex 488nm Em 610/20) and AF647 (Em 633 Ex 660/20) fluorescence was collected as described (Filby et al., 2011) .

**ImageStream acquisition and analysis**

Samples (4 x 10^7^/ml in 60µl of wash buffer with 1μg/ml PI and RNAse A) were acquired on a fully ASSIST calibrated 5-laser 6-Channel ISx Imaging Flow Cytometer with 40x magnification controlled by INSPIRE software (Amnis). Single stained controls were collected with bright-field (BF) illumination off. Samples were acquired with a BF area lower limit of 35μm^2^ to eliminate debris with all necessary excitation laser switched on. A minimum of 50,000 total events was collected per sample. A compensation matrix was created using single stained raw image files (.rif) and the IDEAS 4.0 compensation wizard. The matrix was used to compensate sample .rif files (Table S1). For brevity, full details of all masking, features and analysis strategies are previously described (Filby et al., 2011). Single cell events were identified using the Area and Aspect ratio of Channel 3 (Bright-field) default mask (M03). CTV, PI and pH3 AF488 fluorescence was measured using the Intensity feature and the default M01, M04 and M02 masks respectively. The CTV intensity profile was used to calculate the percent divided and proliferation index as previously(Filby et al., 2007). Mitotic cells were identified by PI and pH3 AF488 intensity. Mask M04 was adapted to subdivide pH3+ events using morphology. The Aspect Ratio and Spot Count were calculated for the new mask and plotted as a bi-variate graph to allow population gating. Telophasic cells were identified as described previously.

**Calculation of division round specific cell cycle precursor frequencies**

The data was analysed as outlined in Figure S2, where the gated divisional frequencies (gF) were first adjusted by 2^n^ where n is the division round (aF). The sum of the aF was set at 100% to then derive the precursor frequency (pF) for each division round. The Watson pragmatic fitting algorithm was used for cell cycle analysis (Watson, Chambers, & Smith, 1987). The reported frequencies of G2/M cells from the model fit were adjusted by the pH3+ gated values to give G2 and M percentages. These frequencies were then used in conjunction with the pF to determine the % of the starting population in a given cell cycle phase within a given division round (cpF).

## Cell Cycle model steps

The steps of the cell cycle model is described as follows:

1. A population of cells are initiated in generation 0 and assigned to a cell-cycle phase. The cPF in each cell-cycle phase is matched to the experimental data.
2. Each cell is randomly assigned a phase-time which is the time since the cell first entered its current phase. Since cells are not synchronized the randomly assigned times are selected from a uniform distribution between 0 and $\mu_{cmp}$.
3. The percentage increase in the number of cells at a later experimental time point is calculated from the cPf of cells in each generation. An estimate of the number of cells that have not transitioned from generation 0 for each phase is made. These cells are assumed to be non-viable for mitosis.
4. From phase at $T=0$, a number of cells corresponding to the estimated number of non-viable cells are randomly chosen to remain in their initial phase for the duration of the simulation.
5. At each time step the global time is incremented. The phase-time of each cell is updated. The failure rate calculation is used to assess the probability of each cell transitioning to the next phase.
6. A random number between 0 and 1 is generated. If the drug blocking routine is activated a second random number between 0 and 1 is generated. If the first random number is greater than the transition probability and the second is greater than the blocking probability for the phase the cell is exiting the cell transitions to the next phase and its phase-time is reset to 0.
7. If cells have transitioned from the M phase to the G_1_ phase and undergone mitosis a daughter cell is created in the G_1_ phase with phase time 0 and in the same generation as its parent.
8. At global time points corresponding to experimental measurement times the cPF of the in-silico population is stored.
9. The simulation ends when the global time is equal to or greater than the experimental time.

## Blocking parameter percentages for treated models

Table S1 percentages of transitions blocked in model fitted to cell populations treated with different cytotoxic agents

| **Treatment** | **% Transitions Blocked from G_1_ phase** | **% Transitions Blocked from S phase** | **% Transitions Blocked from G_2_ phase** | **% Transitions Blocked from M phase** |
| --- | --- | --- | --- | --- |
| **Nocodazole** | <1.0 | 10.2 | 16.2 | 81.1 |
| **Demecolcine** | 10.0 | 27.0 | 17.9 | 63.2 |
| **5-Fluorouracil** | <1.0 | 58.7 | 1.4 | 3.3 |
| **Etoposide** | <1.0 | 61.3 | 99.7 | 95.9 |

## Fitting using differential evolution algorithm

**Background**

Differential evolution (DE) is an iterative, non-gradient based optimization scheme modelled on evolutionary processes (Storn & Price, 1997). A population of randomly initiated vectors, containing potential solutions, are iteratively updated by randomly combining with other members of the population, replacing the original vector solution with the new ‘mutant’ solution vector if it reduces the value of the loss function. Hyper Parameters which can be controlled are, the number of iterations, number of solution vectors in the population, cross over factor, (which gives the percentage of elements of the original vector which will be updated in the mutant vector) and the weight (which weights the contribution of other vectors to the mutant solution).

Hyperparameters for DE fits used in this work are shown in Table S2.

Table S2 Hyperparameters for DE algorithm

| **Hyperparameter** | **Number of iterations** | **Number of solution vectors in population** | **Cross-over factor** | **Weight** |
| --- | --- | --- | --- | --- |
| **Value** | 200 | 25 | 0.9 | 0.5 |

**Fitting our model parameters to the experimental data using DE**

In this work the for every fitting run, we generate 25 vectors of potential solutions, with fit parameters randomly generated from a uniform distribution upper and lower bounded by pre-specified limits (see Table S3). Initially the loss function, comparing the cPF of the experiment with that generated by the model at the experimental measurement time points (32, 48 and 72 hours for the untreated population and at 48 hours for the treated population), is computed for each of the solution population members by running a complete cell cycle model. Following initiation, at each iteration a mutant solution vector is generated for each solution vector. Values of the mutant vectors that fall outside of the predefined limits (Table S3) are replaced with the corresponding value from the parent vector. The loss functions of each mutant vector are evaluated by running a complete cell cycle model, using the mutant solution vectors. If the mutant vector has a lower loss than the parent vector, it replaces the parent in the population. At each iteration the loss reduces toward a global minimum. After the final iteration, the solution vector with the lowest loss is selected as the optimum solution.

The upper and lower bounds on the fit parameters for the untreated cell populations are shown in Table S3

Table S3 Upper and lower limits on fitting parameters for untreated populations

| **Fit parameter** | **G_1_**$\boldsymbol{\mu}^{\boldsymbol{0}}$ | **G_1_**$\boldsymbol{\sigma}^{\boldsymbol{0}}$ | **S**$\boldsymbol{\mu}^{\boldsymbol{0}}$ | **S**$\boldsymbol{\mu}^{\boldsymbol{0}}$ | **G_1_**$\boldsymbol{\mu}^{\boldsymbol{1, 2}}$ | **G_1_**$\boldsymbol{\sigma}^{\boldsymbol{1,2}}$ | **S**$\boldsymbol{\mu}^{\boldsymbol{1, 2}}$ | **S**$\boldsymbol{\sigma}^{\boldsymbol{1, 2}}$ | **G_2_**$\boldsymbol{\mu}^{\boldsymbol{1, 2,3}}$ | **M**$\boldsymbol{\sigma}^{\boldsymbol{1, 2,3}}$ | $\boldsymbol{b}_{\boldsymbol{p}}$ |
| --- | --- | --- | --- | --- | --- | --- | --- | --- | --- | --- | --- |
| **Lower limit** | 2 | 0 | 2 | 0 | 2 | 0 | 2 | 0 | 1 | 0.1 | 0 |
| **Upper limit** | 15 | 3 | 15 | 3 | 15 | 4 | 15 | 4 | 8 | 5 | 1 |

Figure S1 shows how the squared difference error is minimized during a typical fitting run using DE, showing that after 200 iterations there is little improvement in the fit.


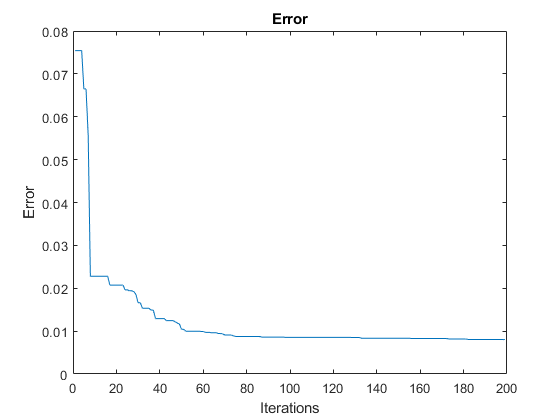


Figure S1 Typical error of fits over 200 iterations (note in minimising the square difference of the cPF we use values between 0 and 1 to represent the percentage, hence the error values appearing small)

## Fits with separate transition function for each phase and generation

Figures S2 and S3 and Table S4 show the results of experiments with the GEV location parameter µ allowed to vary independently for all cell-cycle phases and division rounds. There is good correspondence between the values of µ for each cell-cycle phase in division rounds 1 and 2, prompting the decision to use a single value of µ for each cell-cycle phase across division rounds 1 and 2 in the final model.


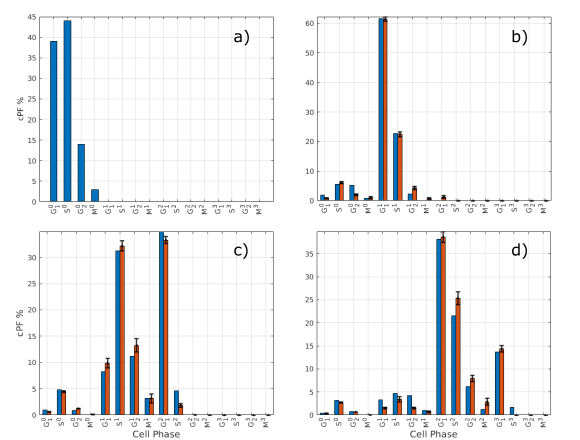


Figure S2 Blue bars show cPF from experimental population and red bars show cPF from in-silico population at a) 0 hours, b) 32 hours, c) 48 hours and d) 72 hours (note at 0 hours cPF of in-silico population is matched to experimental population). Error bars show standard error from fits of 100 in-silico cell populations. Super scripts on x-axis labels indicate cell generation.


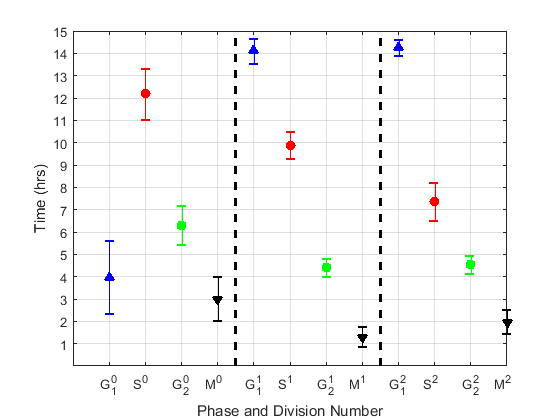


Figure S3 Fitted values of µ for untreated population, errorbars show standard error from 50 fits

| **Division round** | $\boldsymbol{\mu}_{\boldsymbol{G}_{\boldsymbol{1}}}$**(hrs)** | $\boldsymbol{\mu}_{\boldsymbol{S}}$ **(hrs)** | $\boldsymbol{\mu}_{\boldsymbol{G}_{\boldsymbol{2}}}$ **(hrs)** | $\boldsymbol{\mu}_{\boldsymbol{M}}$ **(hrs)** | **Intermitotic**  **Time (hrs)** |
| --- | --- | --- | --- | --- | --- |
| **0** | 3.9 | 12.2 | 6.3 | 3.0 | 25.5 |
| **1** | 14.1 | 9.9 | 4.4 | 1.3 | 29.7 |
| **2** | 14.3 | 7.4 | 4.5 | 1.9 | 28.1 |

Table S4 Mean interphase times and intermitotic for division rounds 1, 2 and 3 obtained from optimizing the cPFs of the in-silico cell model to match experimental results

## Hazard function with GEV vs Normal Distribution

Figure S3 shows typical discrete probabilities of phase transition ($P_{p}^{N}$) for Hazard function applied to a GEV PDF with $\mu$ = 5, $\sigma$ = 0.2 and $\xi$= 0.68. For values of $t \ll\mu$ the probability of transition is 0, so cells must remain in the cell cycle phase for some finite time before transitioning. If $\xi$>0, as $t$ approaches $\mu$, the probability of transition increases, reaching a maximum at $t=\mu$. Therefore $\mu$ is the phase time at which a cell exiting the current phase is most probable. As $t$increases beyond $\mu$, the transition probability decreases exponentially toward 0. For $t > \mu$, the longer the time which cells remain in a phase, the less likely they are to exit the phase during the next time step. This is representative of real cell populations, where cells that remain in a cell-cycle phase for an extended duration become increasingly unlikely to exit the phase and become increasingly likely to be arrested. Using a normal CDF result in a $P_{p}^{N}\to$ 1, so the probability of a cell exiting the phase increases with $t$, until reaching 1, as shown in Figure S4. This is also true for the GEV with $\xi<$0. Clearly this means that cells must exit the current cell phase after a long enough time period, which does not correspond to known cellular behaviour (Zhang, Bassetti, Gherardi, & Lagomarsino, 2017). Also note for both probability distributions, there is a finite time that a cell must remain in a phase before the probability of transition is > 0.


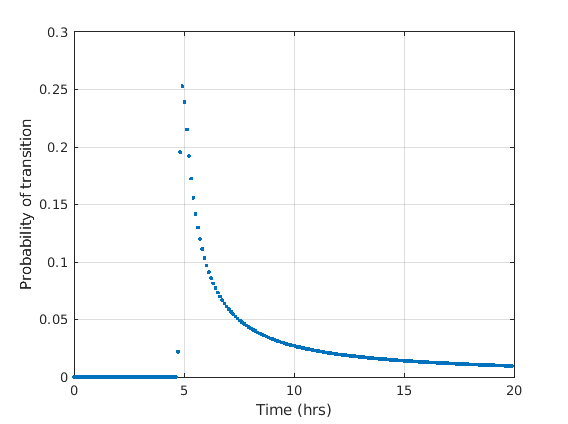


Figure S3 Probability of transition from current phase at discrete time points for Hazard function using GEV CDF (µ = 5, sigma = 0.2, shape = 0.68, time step = 0.1 hours).


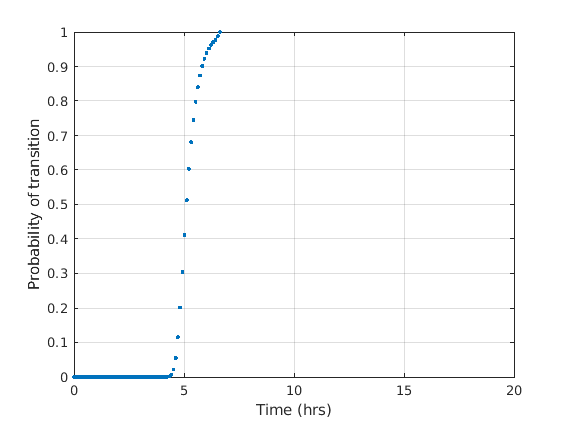


Figure S4 Probability of transition from current phase at discrete time points for Hazard function using GEV CDF (µ = 5, sigma = 0.2, shape = 0.68, time step = 0.1 hours).

## Variation in final fit parameters averaged over iterations

The mean value of the parameters averaged over more than 100 fits effect the final values of the fit parameters by less than 1% for most fit parameters (see Figure S4).

| 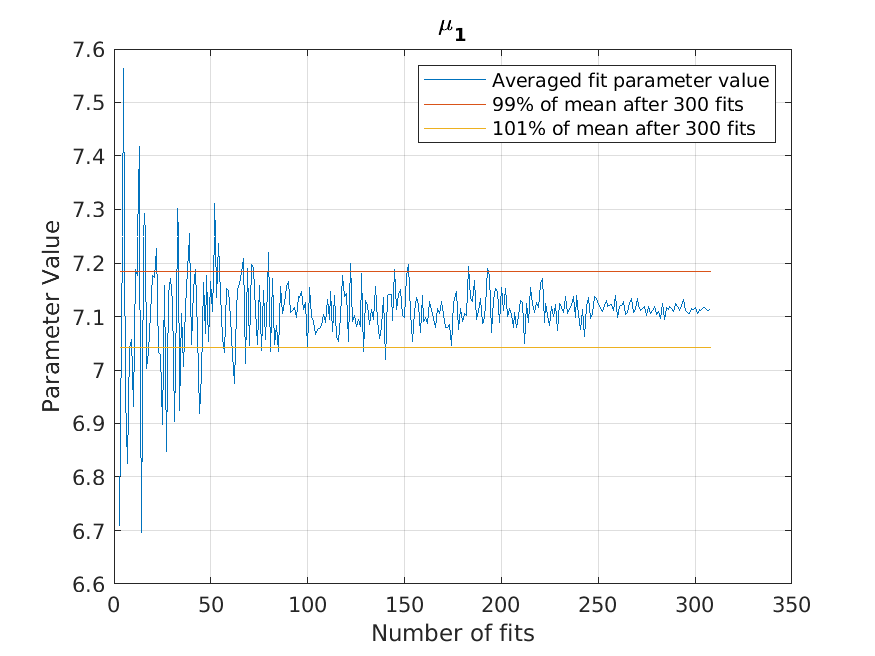 | 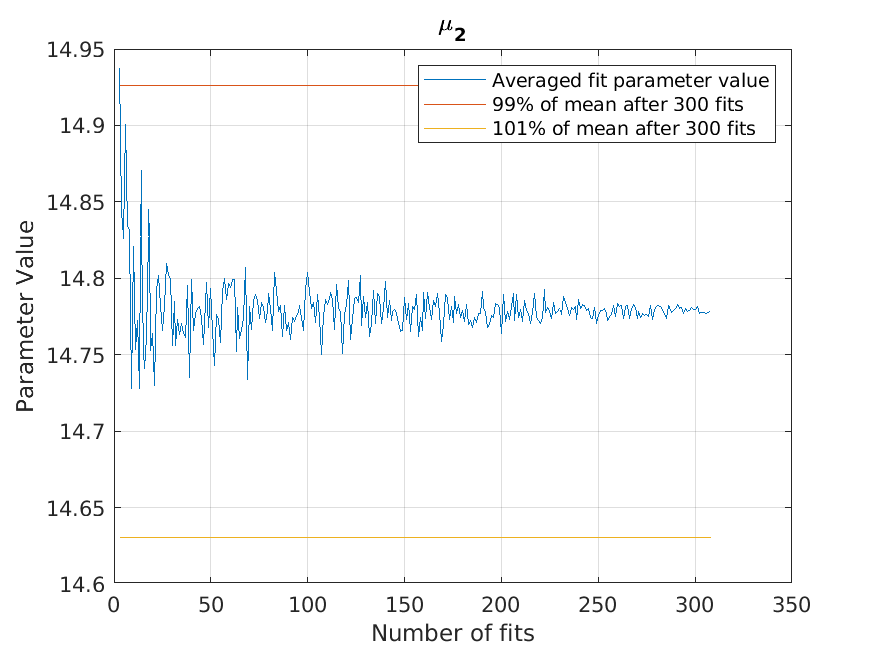 |
| --- | --- |
| 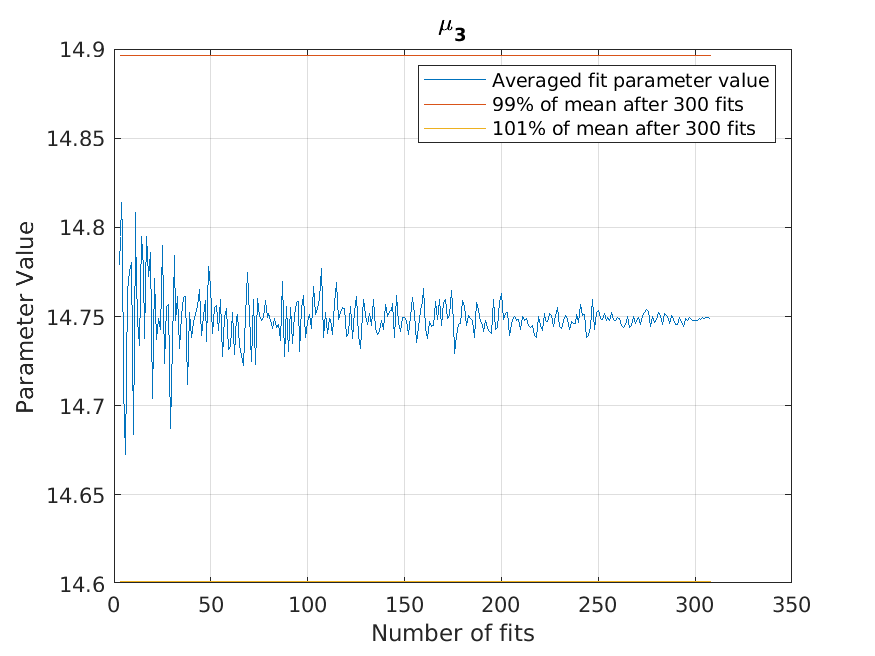 | 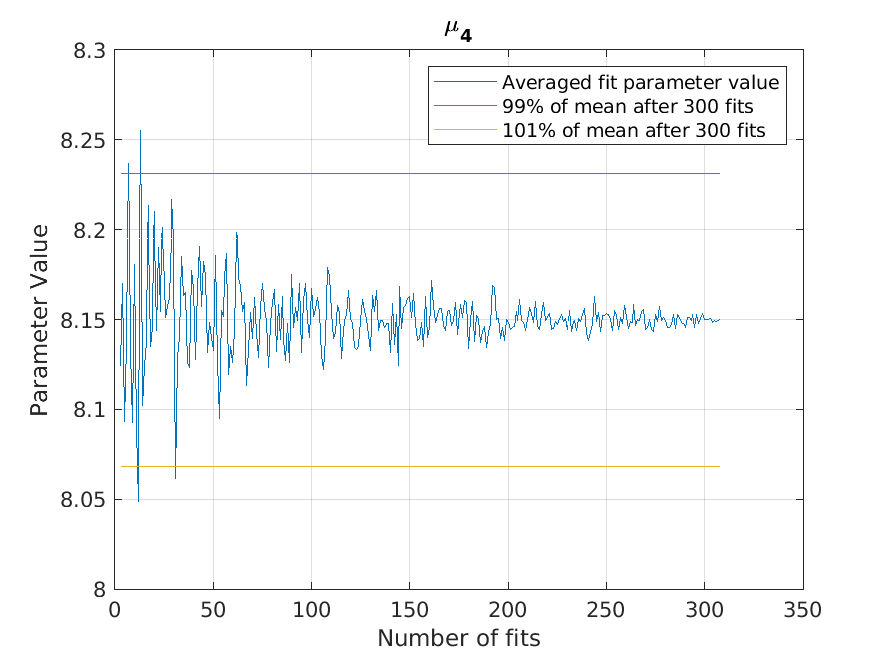 |
| 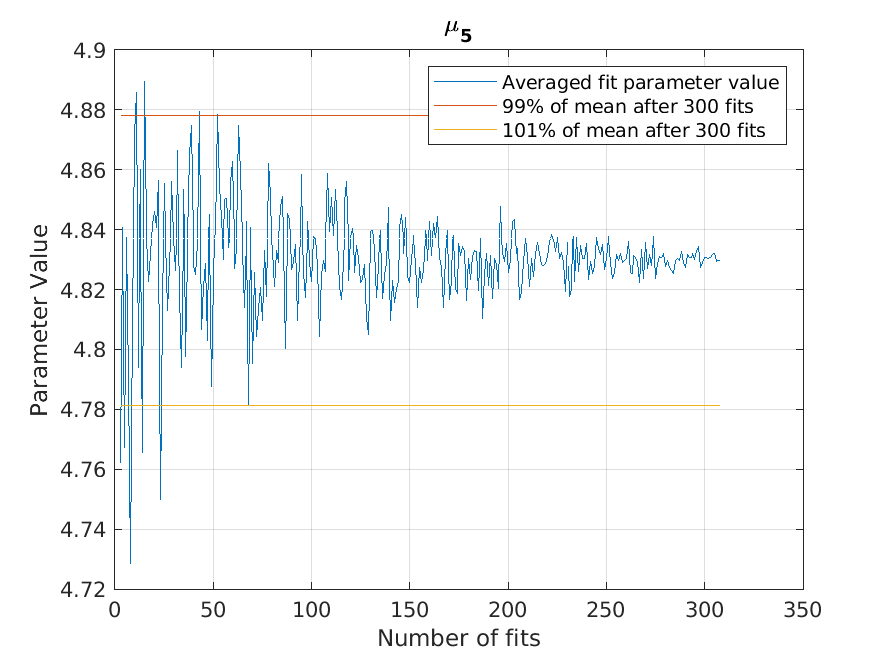 | 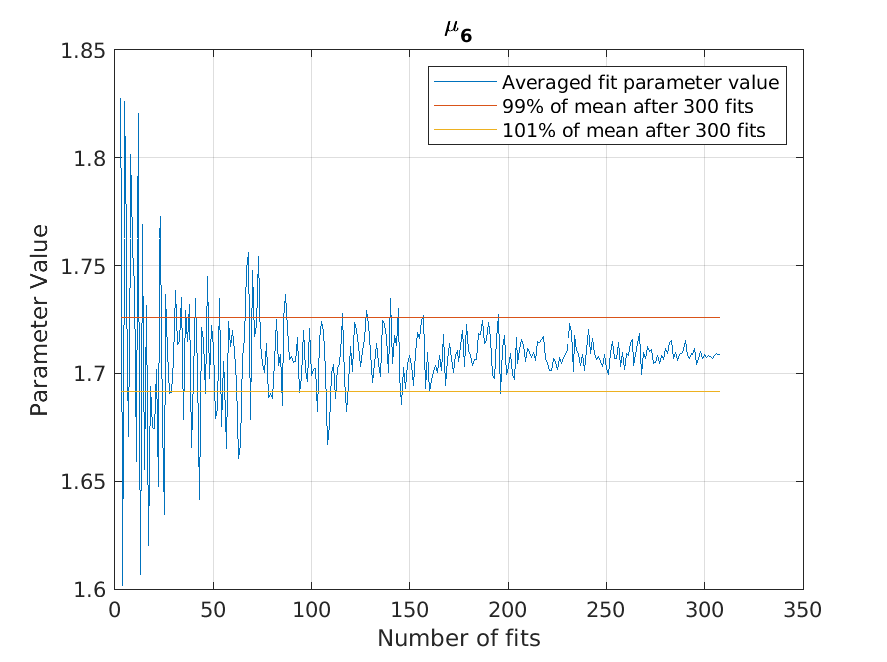 |
| 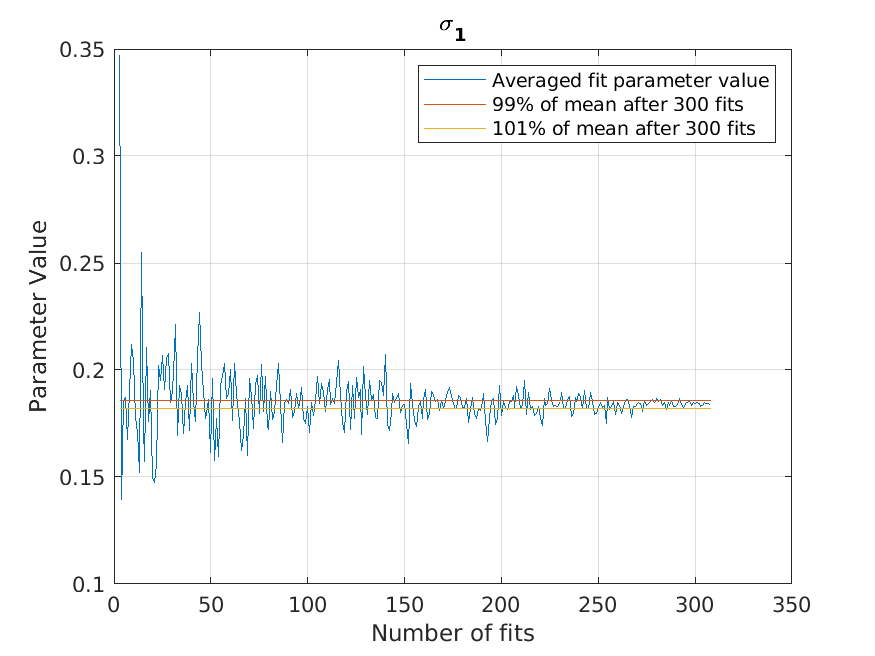 | 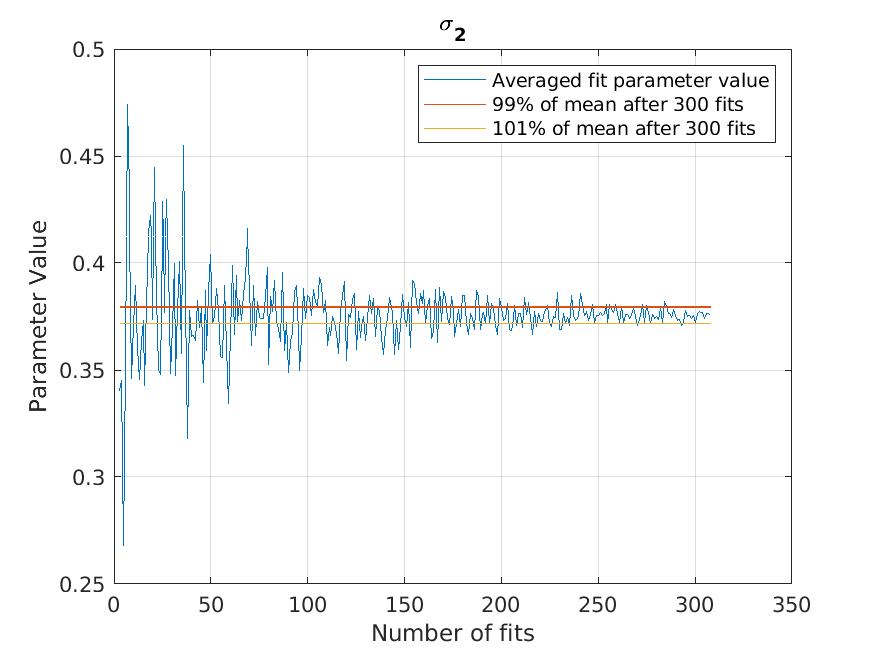 |
| 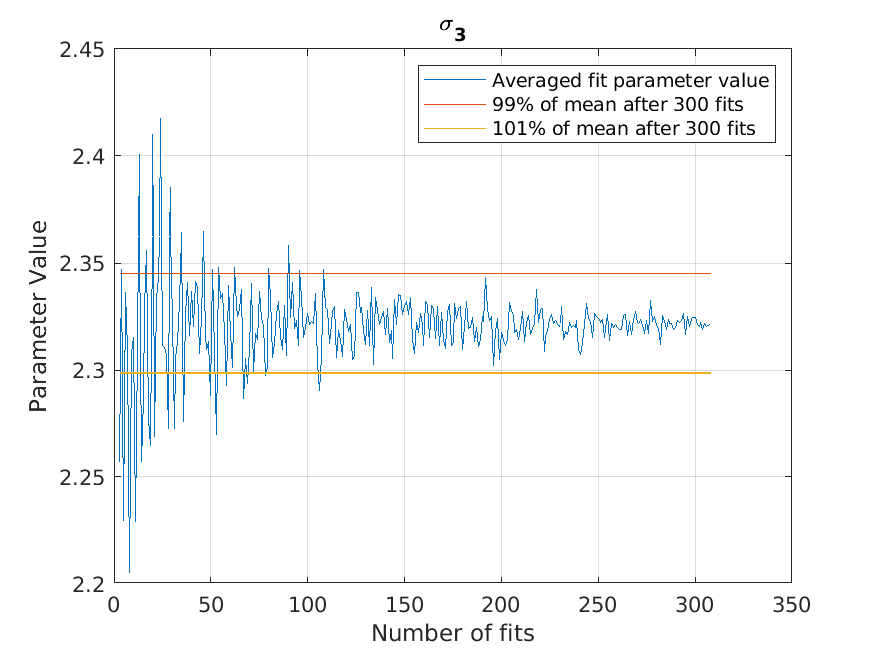 | 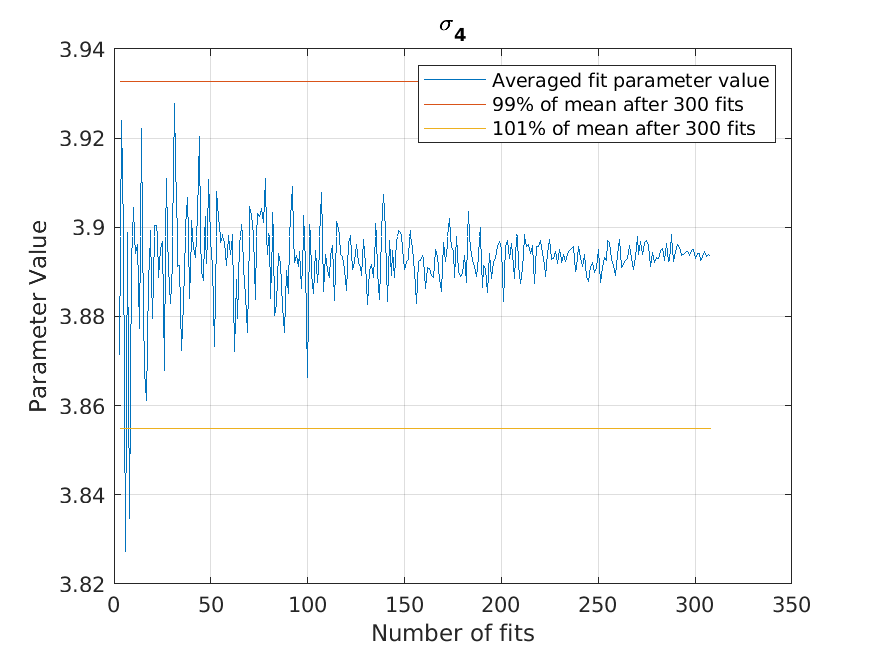 |

Figure 4 Fit parameter values averaged over different numbers of fits. Horizontal red and yellow lines show 99% and 101% of mean after averaging over 300 separate fits.

# References

Filby, A., Perucha, E., Summers, H., Rees, P., Chana, P., Heck, S., … Davies, D. (2011). An imaging flow cytometric method for measuring cell division history and molecular symmetry during mitosis. *Cytometry Part A*, *79A*(7), 496–506. https://doi.org/10.1002/cyto.a.21091

Filby, A., Seddon, B., Kleczkowska, J., Salmond, R., Tomlinson, P., Smida, M., … Zamoyska, R. (2007). Fyn Regulates the Duration of TCR Engagement Needed for Commitment to Effector Function. *The Journal of Immunology*, *179*(7), 4635 LP – 4644. https://doi.org/10.4049/jimmunol.179.7.4635

Storn, R., & Price, K. (1997). Differential Evolution – A Simple and Efficient Heuristic for global Optimization over Continuous Spaces. *Journal of Global Optimization*, *11*(4), 341–359. https://doi.org/10.1023/A:1008202821328

Watson, J. V, Chambers, S. H., & Smith, P. J. (1987). A pragmatic approach to the analysis of DNA histograms with a definable G1 peak. *Cytometry*, *8*(1), 1–8. https://doi.org/10.1002/cyto.990080101

Zhang, Q., Bassetti, F., Gherardi, M., & Lagomarsino, M. C. (2017). Cell-to-cell variability and robustness in S-phase duration from genome replication kinetics. *Nucleic Acids Research*, *45*(14), 8190–8198. https://doi.org/10.1093/nar/gkx556
